# Predictors of Seeking Mental Health Treatment in Black Men: Therapy Fears and Expectations About Counseling

**DOI:** 10.1007/s10597-023-01183-1

**Published:** 2023-09-08

**Authors:** Erlanger A. Turner, Courtland Douglas, Abdul Haseeb

**Affiliations:** 1 Graduate School of Education and Psychology, Pepperdine University, 6100 Center Drive, 5th Floor, Los Angeles, CA 90045 USA; 2 Howard University, Washington, DC USA

**Keywords:** Mental health, Correctional counseling, Help-seeking, Black men, Expectations about treatment

## Abstract

The current study examined predictors of mental health treatment among Black men while incarcerated. Participants were 76 individuals who identified as Black Americans and were recruited from a correctional setting. Using secondary data analysis (Shaw, L. B., & Morgan, R. D. (2011). Inmate attitudes toward treatment: Mental health service utilization and treatment effects. *Law and Human Behavior*, *35*(4), 249–261. 10.1007/s10979-010-9233-5.), results indicated that Black men with a mental health diagnosis were more likely than those without a diagnosis to report receiving mental health treatment while incarcerated. Additionally, linear regression analyses indicated that only expectations about treatment (i.e., personal commitment; not therapy fears or history of mental health diagnosis) significantly predicted the current use of mental health treatment. Implications for research and practice are discussed.

## Predictors of Seeking Mental Health Treatment in Black Men: Therapy Fears and Expectations About Counseling

Scholars note that several predictors of using mental health treatment including help-seeking attitudes, mental health stigma, environmental constraints, affordability of services, and symptoms of distress (e.g., Arria et al., 2011; Corrigan et al., 2012; Turner & Llamas, 2017; Turner, 2019). Among individuals from ethnic and racial groups, research indicates they are significantly less likely to seek mental health treatment (Snowden, 2012; Turner et al., 2016; SAMHSA, 2015). Data collected from the 2019 National Health Interview Survey indicated that non-Hispanic White adults were more likely than non-Hispanic Black adults to have received any mental health treatment (Terlizzi & Zablotsky, 2020). Furthermore, when Black and African Americans use psychiatric treatment, they are more likely to receive services under emergency, mandated, or coerced conditions rather than voluntary conditions (Hu et al., 1991).

Help-seeking attitudes and expectations about therapy also play an important aspect in individuals’ decisions to seek psychological treatment. Decades of research have demonstrated how negative attitudes and perceptions influence Black and African Americans’ decisions to avoid seeking professional help (Masuda et al., 2012; Mishra et al., 2009; Snowden, 2001; 2012; Turner et al., 2016; Wallace & Constantine, 2005). For example, Turner and colleagues (2016) highlighted barriers to Black Americans seeking treatment and noted that, often, individuals may not seek treatment, or they may terminate treatment due to negative attitudes or therapy fears. Studies have shown that men seek help for mental health-related concerns at a lower rate than women (Addis & Mahalik, 2003; Husaini et al., 1994; McKay et al., 1996; Padesky & Hammen, 1981; Thom, 1986). Furthermore, some researchers have reported that expectations about counseling may contribute to mental health services use (Shaw & Morgan, 2011; Tinsley et al., 1994). One study found that those who reported positive expectations about counseling were more actively engaged in the treatment process (Tinsley et al., 1994). However, limited research exists examining counseling expectations among Black men. Taken together, Black men’s mental health and treatment use appears to be a particular concern, and special attention needs be given to treatment use among incarcerated Black men.

### Expectations About Therapy

Among Black men, expectations about treatment may inform help-seeking behaviors or lack thereof. According to the Model of Treatment Initiation (MTI; Turner et al., 2016;, 2019), multiple factors facilitate or inhibit help-seeking among diverse ethnic groups. Thinking further, the model provides insights into expectations regarding psychological services and may clarify the link between treatment expectations and therapy use. The MTI includes four areas: accessibility, availability, appropriateness, and acceptability. Turner and Turner (2021) summarize the MTI as follows: (a) *accessibility* explores multiple factors such as cost, availability of providers of color, and other structural barriers, (b) *availability* explores variables such as the desire for culturally competent services or Black therapists, (c) *appropriateness* includes variables that explain Black people’s preferences regarding healing practices such as working with a therapist versus other sources of help-seeking (e.g., pastoral counseling), and (d) *acceptability* explores variables such as mental health stigma and cultural mistrust. For a more detailed discussion see Turner et al. (2019).

Treatment expectations are also important to understand because they play a vital role in the client’s hope and engagement with different aspects of therapy (Anderson et al., 2013). Shaw and Morgan (2011) note that expectations may include the clients’ views about therapy outcomes or the roles of the client and therapist. In general, some studies have reported that client expectations contributed to therapy success and the client’s engagement (Anderson et al., 2013; Shaw & Morgan, 2011). For example, one early study found that expectations among Black clients was associated with distrust of White therapists (Watkins & Terrell, 1988). According to Watkins and Terrell (1988), when Black clients exhibited higher levels of mistrust, they also expected less from therapy, regardless of the therapists’ race. Additionally, Black and African American men may expect to experience stigma to accompany seeking psychotherapy. In other words, there may be concerns about being negatively labeled as “weak” or “crazy” by oneself or others if they were to begin treatment (Thompson et al., 2004). These general fears about psychotherapy due to its unfamiliarity may be present and intersect with concerns that mental health practitioners will mistreat them similar to how institutions of health have abused Black people in the past (Alang, 2019).

### Therapy Fears and Mental Health

Psychotherapy fears is another barrier that has been shown to impact the use of mental health services. For example, one 2017 study found that psychotherapy fears were a significant predictor of therapy use among college students (Turner et al., 2017). The authors reported that those who reported more fears about therapy were less likely to report a history of seeking services. While the study examined therapy fears among a college student sample, one limitation was that only 6% of the sample self-identified as Black or African American. Fears about possible discrimination on the part of clinicians can also reduce the likelihood of seeking treatment. According to one study, participants endorsed that fear of experiencing double discrimination was a significant barrier to treatment among Black Americans (Alang, 2019). This may significantly reduce individuals’ desires to willingly seek professional help. Taylor and Kuo (2019) note that because of these types of psychotherapy fears, Black Americans are more likely to seek treatment involuntarily or when mandated to attend.

Among Black men, psychotherapy fears may be a particular concern. Studies have consistently shown that men are less likely to seek professional help compared to their counterparts (e.g., Alang, 2019; Turner et al., 2017). Additionally, negative perceptions and psychotherapy fears tend to be more prevalent among men compared to women. For example, men have been found to report less trust, acceptance, genuineness, and tolerance in the counseling relationship compared to women (Kakhnovets, 2011). Among Black and African American men, similar findings have been noted (Vogel et al., 2011; Wallace & Constantine, 2005). According to Vogel and colleagues (2011), Black men endorse a greater degree of dominant masculinity and this may influence feelings of shame around discussing their emotions in therapy. Given what we know to date, it is important that we better understand how perceptions and psychotherapy fears impact the use of mental health services among incarcerated Black men. “Pursuing a clearer understanding of the variables associated with Black Americans’ mental health help-seeking attitudes and intentions can potentially help bridge a critical knowledge and service gap for this population” (p 326; Alang, 2019).

### Mental Health Treatment for Incarcerated Men of Color

Furthermore, according to the Federal Bureau of Prisons (2018), men are incarcerated at a highly disproportionate rate compared to women—over 90% of prisoners are men, while women account for less than 10%. Despite Black Americans making up only 13.6% of the U.S. population, approximately 37% of inmates are Black or African American compared to 58% identified as White (Federal Bureau of Prisons, 2018; Census Bureau, 2021). Among individuals in the criminal justice system, researchers (e.g., Andrews et al., 1990; Baillargeon et al., 2009) have noted that mental health treatment has numerous challenges. Evidence suggests that more than a quarter of inmates have a mental health diagnosis, implying that one of the greatest predictors of incarceration is a mental health ailment (Reingle-Gonzalez & Connell, 2014). One major concern is adequate mental health treatment within jails and prisons. Shaw and Morgan (2011) noted that as the number of individuals in the criminal justice system increased, correctional mental health care providers were becoming more overwhelmed. Furthermore, decisions are not always clear on who should receive mental health treatment while incarcerated and how many sessions are appropriate (Andrews et al., 1990).

Despite the fact that African Americans and Whites in Jail experience similar rates of mental illness, disparities in treatment become apparent in the community before their arrest. In a study investigating mental health treatment seeking prior and during incarceration, results found that African American jail inmates are less likely to report having received mental health treatment prior to arrest, in contrast to Whites (Youman et al., 2010). However, once incarcerated, both African American and White jail inmates exhibit comparable levels of utilizations of mental health treatment when provided with access to treatment (Youman et al., 2010; Meyer et al., 2014). Within the correctional system, the playing field for incarcerated individuals are more so leveled between the races, making it more apparent of the disparities that exist in the community due to systemic barriers and a lack of resources for men of color (Youman et al., 2010). With that in mind, it would be reasonable to suggest that for men of color, disparities in treatment continue to exist when a formerly incarcerated person reintegrates into the community when the same pre-existing barriers in accessing treatment persist and potentially are even further compounded by the stigma associated with incarceration (Mahaffey et al., 2018).

The mental health treatment of those incarcerated is a significant concern that deserves attention. One large-scale study using a sample of 79,000 inmates in the U.S. found that inmates with psychiatric disorders were more likely to also have had prior incarcerations, in contrast to those without a diagnosis of a mental illness. According to the authors, inmates previously diagnosed with a mental health disorder were more likely to recidivate due to inadequate care or limited capacity by the legal system to identify mental health problems resulting in placements between the criminal justice system and hospitalization (Baillargeon et al., 2009, p. 106). The major implication here is that inmates diagnosed with a psychiatric disorder are not receiving adequate treatment while incarcerated. This may lead to difficulties with adjustment to society after being released.

### Purpose of the Present Study

The purpose of the current study is to examine perceptions about mental health and treatment use among Black men in a correctional setting. Using secondary data analysis, we were interested in understanding how perceptions and fears about psychotherapy predicted the use of mental health services among this population. The following research questions were examined: (a) whether individuals with a history of a mental health diagnosis will be more likely to report current mental health treatment while incarcerated, and (b) whether fears of therapy, mental health diagnosis, and expectations about treatment will predict current mental health services use among incarcerated Black men.

## Method

### Participants

Participants were 76 men who self-identified as Black or African American recruited from a correctional facility in the Midwestern region of the US. Participants’ ages ranged from 19 to 54 years old (*M* = 31.64, *SD* = 8.67). For educational background, 26% received a GED, 15% earned a high school diploma, 7% earned a vocational or trade certification, 3% earned an Associate’s degree, 1% a Bachelor’s degree, 18% had no diploma, and 30% did not report their educational level. Individuals were incarcerated for multiple violent crimes (27.6%), multiple non-violent crimes (15.8%), robbery (17.1%), drugs or alcohol distribution (13.2%) or other crimes, such as homicide, sex crimes, or property crimes (26.3%). 21% of participants were identified as having a mental health diagnosis. Of the total sample, 22% reported currently receiving individual or group psychotherapy and 45% reported previously receiving mental health treatment while incarcerated. Participants reported being mandated (7.9%) to therapy for numerous issues such as depression (11.8%), substance disorders (6.6%), anger management (6.6%), anxiety (5.3%), and other difficulties (69.7%). The remaining participants reported voluntary treatment use (21.1%) or did not indicate a response (71%). A summary of the demographic features of this sample is presented in Table 1.

**Table 1:** Summary of the demographics features of the sample (*N* = 76)

Highest Degree Earned	*n* (%)
High school	11 (15)
GED	20 (26)
Vocational	5 (7)
Associates Degree	2 (3)
Bachelor’s Degree	1 (1)
No diploma	14 (18)
Unknown	23 (30)
Marital Status	
Single	39 (51)
Married	9 (12)
Divorced/Separated	5 (7)
Partnered	10 (13)
Not reported	13 (17)
Mental Health Treatment Use	
Past treatment use	34 (45)
Current treatment use	17 (22)
Reason for Treatment	
Schizophrenia	1 (1)
Depression	9 (12)
Anxiety	4 (5)
Substance disorder	5 (6)
Anger management	5 (7)
To talk	2 (2)
Program agreement	1 (1)
Family problems	1 (1)
Relationship issues	1 (1)
Medical condition	1 (1)
Not reported	46 (61)

### Measures

#### Demographic Information

To gather background information, participants completed a demographic form on the following: age, ethnicity, education level, and reason for incarceration. Researchers (Shaw & Morgan, 2011) accessed the mental health database to review participants’ mental health records to obtain information regarding diagnosis and treatment utilization. However, no specific types or modalities of treatment were reported.

#### Therapy Fears

To assess fears of seeking therapy, participants completed the Thoughts About Psychotherapy Survey (TAPS; Kushner & Sher, 1989). The TAPS is a global measure of fears about seeking mental health services. Participants are presented with a list of 19 various fears on a Likert-scale rated from 1 (*no concerns*) to 5 (*very concerned*). Sample items include, “whether the therapist will share my values,” “whether my friends will think I’m abnormal for going to therapy,” and “whether everything I say in therapy will be kept confidential.” The ratings are summed across all items. The TAPS total score can range from 19 to 95, with higher numbers indicating greater therapy fears. The TAPS scores have demonstrated strong reliability (e.g., ranging from 0.89 to 0.94) across numerous studies (Deane & Chamberlain, 1994; Turner & Llamas, 2017; Turner, 2018). Studies have also noted discriminant and convergent validity of the TAPS (Deane & Chamberlain, 1994; Kushner & Sher, 1989). The Cronbach’s alpha was 0.95 for the current sample.

#### Expectations About Counseling

To measure inmates’ expectations of seeking mental health treatment, the Personal Commitment subscale of the Expectations About Counseling Questionnaire–Brief Form (EAC-B; Tinsley, 1982) was used. The Personal Commitment subscale consists of 18 items rated on a Likert-scale ranging from 0 (*not true*) to 7 (*definitely true*) to assess participants’ expectation of on-going involvement in psychotherapy. Sample items include, “expect to discuss my concerns”, and “expect to express and discuss feelings.” Ratings are summed for a subscale score, with higher scores on the EAC-B indicative of the willingness to participate and actively engage in treatment. Studies have supported reliability of the EAC-B scores with alpha coefficients ranging from 0.82 to 0.95 (Anderson et al., 2013; Shaw & Morgan, 2011). Furthermore, psychometric studies have demonstrated the factor structure of the measure (Tinsley et al., 1994; Anderson et al., 2013), as well as convergent and discriminant validity (Tinsley et al., 1994; Tinsley & Westcot, 1990). The Cronbach’s alpha on the Commitment subscale scores was 0.94 for the current sample.

### Procedure

This study was completed using secondary data analyses with the approval of the researchers from Texas Tech University (Lucas B. Shaw and Robert D. Morgan). Data was originally collected through correctional facilities located in the Midwestern region of the US. Inmates were greeted by one of the researchers and informed of the nature and purpose of this study. Participation was on a volunteer basis and all study measures were in English. Inmates that agreed to participate in the study were provided details about their participation and then completed an informed consent form. All participants were then instructed to complete the measures in the questionnaire packet. The measures were organized based on a Latin square design to reduce test order effects (Richardson, 2018). Once an inmate’s questionnaire packet was complete, researchers accessed the mental health database to review participants’ mental health records to determine the number of mental health treatment sessions received in the correctional system. For a complete description of the recruitment and procedures, refer to the larger study that was previously published on inmate attitudes towards treatment and mental health services utilization (see Shaw & Morgan, 2011). The appropriate Institutional Review Boards approved the study under the direction of the primary researchers.

## Results

### Data Analyses

Descriptive and preliminary analyses were conducted to screen for assumptions of normality, linearity, and multicollinearity. No assumptions were violated. None of the major continuous variables were skewed according to recommended cutoff values of 2 for skewness and 7 for kurtosis (West et al., 1995). Furthermore, no outliers were detected based on the frequencies and distribution of the major continuous variables (Barnett & Lewis, 1994). Pairwise deletions were used in analyses containing some missing data. Table 2 displays the mean and standard deviations for the major study variables.

**Table 2:** Descriptive statistics and correlations for study variables

Variable	*M*	*SD*	Range
1. Therapy Fears	49.02	20.53	19–95
2. Expectations About Therapy	88.06	23.45	34–126
3. Age	31.64	8.67	19–54

Note. *N* = 76. Therapy fears was measured using the TAPS (higher scores = more concerns about using therapy; Expectations about therapy was measured using the Personal Commitment scale of the EAC-B (higher scores = willingness to engage in treatment)

### Primary Analyses

An independent sample chi-square test was performed to examine the relation between mental health diagnosis (diagnosis versus no diagnosis) and treatment status while incarcerated (treatment versus no treatment). Chi-square analysis indicated a statistically significant difference (*X*^2^ [*df* = 1] = 18.41, *p* < .0001). Effect size as measured by Cramer’s *V* was 0.49. Black men who were diagnosed with a mental health disorder (21.3%) were more likely to report currently receiving treatment (i.e., individual or group psychotherapy) than those who received no mental health diagnosis (78%).

Linear regression was performed to examine the role of therapy fears, expectations about treatment, and mental health diagnosis on predicting current mental health treatment use among incarcerated Black men. Regression analyses indicated statistical significance [*F*(3, 65) = 13.65, *p* < .0001, *R*^2^ = 0.39] for current mental health use. However, only mental health diagnosis [*t*(68) = 5.88, *p* < .0001, *β* = 0.65, *SE* = 0.11] and level of expectations about personal commitment to therapy [*t*(68) = 1.93, *p* = .05, *β* = 0.003, *SE* = 0.002] were significant predictors of the use of mental health treatment. Black men who were diagnosed with a mental health issue were more likely to currently be engaged in treatment than those who did not have a mental health diagnosis. Also, Black men who reported higher levels of personal commitment to engage in treatment were more likely to be currently attending mental health treatment while incarcerated.

## Discussion

Using secondary data analysis, the primary goal of this study was to examine the relationships between perceptions about mental health and treatment use among Black men in a correctional setting. Our first research question was to examine whether Black men with a history of a mental health diagnosis would be more likely to report the use of current treatment while incarcerated. Our finding supported this hypothesis. Results indicated that Black men with a mental health diagnosis were more likely to report a history of receiving mental health treatment while incarcerated compared to those without a diagnosis. Previous studies have consistently demonstrated that Black Americans and men are less likely to utilize mental health services compared to other ethnic and racial groups and women (e.g., Cheng et al., 2013; Snowden, 2001; Turner et al., 2016; Vogel et al., 2011). Within the criminal justice system, one study found significant racial and ethnic differences in access to mental health treatment (Han & Redlich, 2018). Han and Redlich (2018) reported that African Americans were less likely to access both mental health and substance abuse services than White Americans even though both were sentenced by the mental health courts. Conversely, our findings noted that Black men within a correctional setting were more likely to receive treatment when they reported a history of a mental health diagnosis. One explanation for this finding is that inmates were more likely to receive care due to fewer barriers accessing services compared to outpatient treatment. Similar conclusions were found based on a study examining psychological service use between White and African American inmates. Specifically, Youman and colleagues (2010) noted that systemic barriers and lack of community resources, rather than psychocultural barriers, may be more prominent in explaining African Americans’ disparities in seeking treatment. This is consistent with the MTI which postulates that accessibility variables such as cost and availability of providers may serve as a barrier to care (Turner, 2019). Given that treatment is more accessible to Black men within a correctional setting compared to when in the community, this could explain our findings. By reducing structural barriers such as cost and traveling to an appointment to receive treatment may have been critical to improving access to treatment for those with a mental health diagnosis.

Another possible explanation to consider regarding why incarcerated Black men with diagnoses were significantly more likely than those without diagnoses to report a history of mental health treatment while incarcerated is their recognition of need. Though incarcerated African American/Black men with and without diagnoses had access to these services while incarcerated, recognition of need for treatment may have been the deciding factor in choosing to utilize mental health services. Men without diagnoses may have seen mental health services as unnecessary due to an absence of symptoms, experiencing below threshold symptoms, or experiencing minimal distress and impairment in their functioning. It should be noted that the men in the sample may have received their diagnosis for the first time while incarcerated. Those who met criteria for diagnoses prior to incarceration but were not formally diagnosed until being incarcerated may have been motivated to undergo treatment upon learning about their diagnosis and were able to do so since it was accessible. Within the MTI (Turner et al., 2016), appropriateness factors (e.g., preferences for working with a therapist versus seeking help from friends/family) can contribute to treatment seeking. It is possible that by having a diagnosis individuals were more likely to perceive treatment as being appropriate.

Our results partially supported our second research question – regarding the role of fears of therapy, mental health diagnosis, and expectations about the use of mental health services while incarcerated. Specifically, the data indicated that only mental health diagnosis and expectations about therapy predicted current treatment use. Previous research has indicated that MTI acceptability variables such as therapy fears negatively predicted mental health use (e.g., Deane & Chamberlain, 1994; Shaw & Morgan, 2011; Turner et al., 2017). However, therapy fears were not a significant predictor in the current study. It is possible that because treatment was frequently mandated, therapy fears did not impact decisions to seek treatment. Only 20% of the current sample indicated voluntary treatment. According to one previous study, increased treatment use among inmates may have facilitated increased disclosure and increased concerns about seeking psychological treatment (Shaw & Morgan, 2011).

Additionally, some research suggest that provision of psychoeducation is related to recognition of need, sometimes acting as a precursor to understanding the implications of one’s diagnosis and willingness to seek treatment. Based on data analyzed in this study, it is unclear whether diagnosed individuals were provided psychoeducation on their condition. However, it is common that psychoeducation is provided as part of diagnostic feedback. If these men were provided psychoeducation, the greater number of incarcerated Black men with diagnoses who used mental health services may also be accounted for by this factor. A meta-analysis conducted supports this possibility as they found that men were generally more likely to engage in help-seeking and have fewer fears about therapy following psychoeducation (Sagar-Ouriaghli et al., 2019). The mechanisms by which psychoeducation motivates help-seeking are many but important to note are those components which promote accurate understanding of mental health disorders and normalization of experience. These aspects may be particularly important for Black men, who may be concerned with mental health-associated stigma (Ward et al., 2013). With the aforementioned components in mind, it is possible that psychoeducation imbued these men with knowledge about their diagnoses and how symptoms may have been/are affecting them. Consistent with the MTI, acceptability factors including stigma and therapy fears often service as a barrier to treatment use (Turner, 2019).

Additionally, our study explored the role of expectations about treatment. While studies have shown that men tend to be less comfortable attending counseling (Kakhnovets, 2011), other studies have shown that the race of the counselor or therapist may influence expectations about treatment use (Alang, 2019). In the current study, Black men were more likely to receive mental health treatment while incarcerated when they endorsed a personal commitment to be actively engaged in treatment compared to those who endorsed lower levels of personal commitment. Consistent with the MTI, appropriateness variable such as expectations or willingness to express and discuss feelings can impact treatment use (Turner, 2019). In the current study, inmates commitment to working with a therapist appeared to predict use of treatment. In general, our findings are similar to other studies in correctional settings regarding the use of mental health services (Youman et al., 2010).

### Limitations

This preliminary study sheds some light on mental health use among Black men within a correctional setting. However, some limitations exist. First, we used secondary data, limiting the measures used and thus our ability to examine certain relations among variables of interest. Furthermore, the sample size was small and some participants were excluded from the analyses due to missing data. The limited sample size may have resulted in insignificant findings related to therapy fears, as there may have been insufficient power to detect a meaningful difference. It is possible that with a larger and more comprehensive data set we could have been able to capture a better picture of mental health use among Black men who were incarcerated.

It would have also been useful to understand the specific types of treatments that inmates received. However, due to the nature of the study using archival data we were unable to explore this question. Based on the data, no information appeared to be obtained regarding treatment other than treatment utilization and number of sessions attended. Previous studies on this population found that the more infractions the inmate exhibited was associated with attending more session (Shaw & Morgan, 2011). Black men who are incarcerated may have limited ability to make decisions about treatment use because mental health treatment is not always optional. Often individual or group therapy may be mandated which may also have contributed to our findings about those with a diagnosis being more likely to receive treatment while incarcerated.

### Future Directions and Implications

To better understand how context shapes Black men’s use of therapy, studies are needed to examine differences between treatment offered in the criminal justice system and within the community. Additionally, future research should be conducted to better understand how cultural attitudes (e.g., ethnic identity) and masculinity norms, may influence mental health use among incarcerated men and those within the community. The majority of research about men, regardless of their ethnic background, has demonstrated that men are less likely to express a willingness to seek therapy (e.g., Mahalik et al., 2003; Vogel et al., 2011). Furthermore, Pleck (1995) note that these discrepancies between behaviors versus expectations set by traditional masculinity ideology may shape decisions whether to use or delay seeking mental health services. Future research should also examine how levels of masculine ideology may influence seeking mental health treatment. However, the current data did not measure masculinity norms which limited out ability to explore this connection.

An intriguing avenue for further investigation would be to examine the satisfaction levels of inmates with respect to their access to treatment and the quality of the treatment itself, encompassing both during their time within correctional facilities and after their discharge. This could provide valuable insights into the extent to which programs meet their needs and expectations as well as whether men of differing ethnic backgrounds have different needs, particularly Black men. By examining their satisfaction levels, areas for improvement can be identified to enhance the overall quality of interventions. It would also be worthwhile to conduct an evaluation of the appropriateness of the treatment provided, assessing the extent to which it aligns with current evidence-based practices. By examining the adherence to evidence-based approaches, researchers can identify programs and treatments that are not up to standard and refine interventions to specifically optimize mental health treatment in correctional facilities. Exploring the impact of treatment on recidivism rates could also be key in understanding its long-term effectiveness in reducing reoffending. Investigating the relationship between the type and intensity of treatment received may shed light on the efficacy of different interventions and their potential for inmates’ successful reintegration into society. To further gain an understanding of these dynamics, future research could explore the availability and utilization of evidence-based practices in both correctional facilities and the community. Understanding the impact of evidence-based treatment provision on inmates’ perceptions and engagement with rehabilitative programs can inform strategies to enhance treatment effectiveness and promote positive attitudes towards interventions, where men of color on average report more negatively on. These attitudes can then be measured as another potential avenue to address the mental health treatment gap that exists today.

Despite the limitations, our findings suggest that Black men diagnosed with a mental health condition often receive treatment while incarcerated. These findings provide some hope that when individuals have a diagnosis it increases the likelihood of these individuals receiving mental health treatment, thereby reducing the mental health challenges in and out of prison and potentially reducing recidivism rates among this group. For clinicians providing treatment within the criminal justice system, it is important to explore Black men’s expectations about therapy at the beginning of the therapy relationship. Based on our findings, if men endorse low personal commitment towards therapy, it may be more difficult for them to start therapy and establish a working relationship to in order to meet their mental health concerns. Exploring therapy expectations and commitment may allow for clinicians to address this barrier through psychoeducation which could enhance treatment engagement.
